# The role of CMR in the diagnosis of systemic vasculitis: a case study

**DOI:** 10.1186/1532-429X-11-S1-T8

**Published:** 2009-01-28

**Authors:** Phillip Andrews

**Affiliations:** University Hospital of North Staffordshire, Stoke-on-Trent, UK

**Keywords:** Vasculitis, Cardiac Magnetic Resonance, Steady State Free Precession, Left Ventricular Thrombus, Ventricular Thrombus

An 18 yr old caucasian man presented with neurological symptoms and breathlessness. Echocardiography demonstrated a left ventricular abnormality of unclear aetiology. The patient was referred onwards to our unit for further assessment by cardiac magnetic resonance. Imaging was performed on a 1.5 T Philips Intera Acheiva, using a five element phased-array surface coil.

On steady state free precession (SSFP) cine images the appearances were of marked left ventricular hypertrophy with partial cavity obliteration. Left ventricular cavity volumes were reduced and ejection fraction was 54%. Early post contrast inversion recovery gradient echo (IRGE) images showed an area of low signal within the LV cavity distinct from the myocardium. The mass was markedly hypointense to both myocardium and the blood pool. Delayed IRGE sequences 10 minutes following the administration of contrast demonstrated widespread subendocardial hyperenhancement in all myocardial segments sparing only the basal septum. CMR was able to confidently establish the diagnosis of endomyocardial fibrosis with a large left ventricular thrombus burden.

The patient had no history of foreign travel or malnutrition, making a vasculitis the most likely aetiology.

Baseline blood testing demonstrated a mild eosinophilia of 17%, a C reactive protein of 27 mg/L (normal 0–5) and an erythrocyte sedimentation rate of 27 mm/h (normal 0–20). Further imaging demonstrated normal renal arteries on MR angiogram. However CT thorax demonstrated pulmonary haemorrhage and MRI brain demonstrated white matter abnormalities in the cerebral cortex. These findings were in keeping with the clinical diagnosis of vasculitis, Although autoantibodies were all normal, the most likely process is a variant of Churg Strauss.

Cardiac MR was able to establish the diagnosis of endomyocardial fibrosis which was pivotal in identifying the underlying vasculitic disease process.

